# BDNF regulates atypical PKC at spinal synapses to initiate and maintain a centralized chronic pain state

**DOI:** 10.1186/1744-8069-9-12

**Published:** 2013-03-20

**Authors:** Ohannes K Melemedjian, Dipti V Tillu, Marina N Asiedu, Edward K Mandell, Jamie K Moy, Victoria M Blute, Caleb J Taylor, Sourav Ghosh, Theodore J Price

**Affiliations:** 1 Department of Pharmacology, The University of Arizona School of Medicine, Tucson, USA; 2 Department of Cellular and Molecular Medicine, The University of Arizona School of Medicine, Tucson, USA; 3 Bio5 Institute, University of Arizona, Tucson, USA; 4 Graduate Interdisciplinary Program in Neuroscience, University of Arizona, Tucson, USA

## Abstract

**Background:**

Chronic pain is an important medical problem affecting hundreds of millions of people worldwide. Mechanisms underlying the maintenance of chronic pain states are poorly understood but the elucidation of such mechanisms have the potential to reveal novel therapeutics capable of reversing a chronic pain state. We have recently shown that the maintenance of a chronic pain state is dependent on an atypical PKC, PKMζ, but the mechanisms involved in controlling PKMζ in chronic pain are completely unknown. Here we have tested the hypothesis that brain derived neurotrophic factor (BDNF) regulates PKMζ, and possibly other aPKCs, to maintain a centralized chronic pain state.

**Results:**

We first demonstrate that although other kinases play a role in the initiation of persistent nociceptive sensitization, they are not involved in the maintenance of this chronic pain state indicating that a ZIP-reversible process is responsible for the maintenance of persistent sensitization. We further show that BDNF plays a critical role in initiating and maintaining persistent nociceptive sensitization and that this occurs via a ZIP-reversible process. Moreover, at spinal synapses, BDNF controls PKMζ and PKCλ nascent synthesis via mTORC1 and BDNF enhances PKMζ phosphorylaton. Finally, we show that BDNF signaling to PKMζ and PKCλ is conserved across CNS synapses demonstrating molecular links between pain and memory mechanisms.

**Conclusions:**

Hence, BDNF is a key regulator of aPKC synthesis and phosphorylation and an essential mediator of the maintenance of a centralized chronic pain state. These findings point to BDNF regulation of aPKC as a potential therapeutic target for the permanent reversal of a chronic pain state.

## Background

How acute injury transforms to chronic pain remains a long-standing, unresolved question with important medical ramifications. The natural history of most chronic pain conditions suggests that achieving clinically meaningful endpoints requires interventions aimed at targeting or reversing pathological changes that maintain sensitization in these chronic pain states. While studies on plasticity of sensory neurons and CNS structures after injury have led to a wealth of molecular targets implicated in the initiation of pain in preclinical models [1-6], our understanding of molecular mechanisms that maintain chronic pain states remains poor.

Recent advances in understanding how neural circuits maintain long-lasting plasticity may offer insights into how pain becomes chronic [5,6]. Analogous to pain, the encoding of memory engrams in CNS structures is separated into initiation and maintenance phases. Initiation of engram encoding requires protein synthesis [7] and an atypical protein kinase C (aPKC) called PKMζ [8]. Maintenance of the engram is has been linked to PKMζ as PKMζ represents the only known kinase whose activity is required for the maintenance of late-long-term potentiation (LTP) and long-term memory [8], although recent studies have called this hypothesis into question [9,10]. We have demonstrated that the pharmacology and molecular mechanism of a chronic pain state in mice parallels memory engram encoding in the CNS wherein the maintenance phase is critically dependent on PKMζ [11]. These findings have been expanded upon by several groups [12-15] showing that spinal PKMζ is a crucial kinase for the maintenance of pain states that are no longer dependent on afferent input [13]. This conclusion is supported by a lack of effect of spinal PKMζ inhibitors in peripheral nerve injury models wherein afferent input is continuous as a result of the nerve injury [12,15]. On the other hand, following peripheral nerve injury, PKMζ in other CNS regions such as the anterior cingulate cortex, plays a key role in spontaneous pain evoked by injury [12,15].

Hence, PKMζ, and possibly other aPKCs, are key targets for the maintenance of chronic pain states and for the maintenance of long-term memory; however, remarkably little is known about how PKMζ is regulated at CNS synapses. Even less is known about the regulation of other aPKCs, such as PKCλ in the CNS. The importance of this gap in knowledge is driven home by recent controversy in the field wherein the use of ZIP as a specific PKMζ inhibitor has been called into question [9,10]. Brain-derived neurotrophic factor (BDNF), like PKMζ, plays a key role in the initiation and maintenance of LTP and long-term memories [16] and is an important mediator of pain in the dorsal horn [17-21]. Hence, we hypothesized that BDNF, via its receptor: tyrosine receptor kinase type B (trkB), might play an important role in regulating PKMζ and possibly other aPKCs. Our findings indicate that BDNF stimulates PKMζ phosphorylation and synthesis of PKMζ and PKCλ via activation of PDK1/AKT/mTOR signaling at spinal and cortical synapses. Moreover, we show that BDNF is required for the initiation and maintenance of a chronic pain state strongly implicating a BDNF/aPKC signaling module as a key regulator of centralized chronic pain. Therefore, we have elucidated the first neurotransmitter/neurotrophin involved in spinal, synaptic aPKC regulation and linked this system to the initiation and maintenance of a central engram encoding a chronic pain state.

## Results

### Maintenance of persistent sensitization is independent of CaMKIIα and MEK/ERK signaling

We have previously used a model of persistent sensitization, based on rat models of hyperalgesic priming [22], to demonstrate a role for PKMζ in maintenance of a chronic pain state [11]. A key feature of this model is that after the resolution of an initial allodynic state, a subsequent nociceptive hypersensitivity can be revealed by hindpaw injection of a normally subthreshold dose of prostaglandin E_2_ (PGE_2_), causing a prolonged allodynia, or spinal administration of the mGluR1/5 agonist DHPG, causing pronounced nocifensive behaviors [11,22]. In naïve animals, PGE_2_ and DHPG only elicit transient allodynia or nocifensive behaviors, respectively. Hence, this model establishes a persistent sensitization that can be clearly divided into an initiation (induction of priming with interleukin 6 (IL-6)) and maintenance phase (PGE_2_) that persists for long periods of time. Consistent with concepts governing memory encoding and the pharmacology of LTP, our previous findings demonstrate that persistent nociceptive sensitization initiation requires spinal protein synthesis and is reversible by the aPKC inhibitor ZIP whereas maintenance is solely dependent on ZIP reversible process [11].

**Figure 1:**
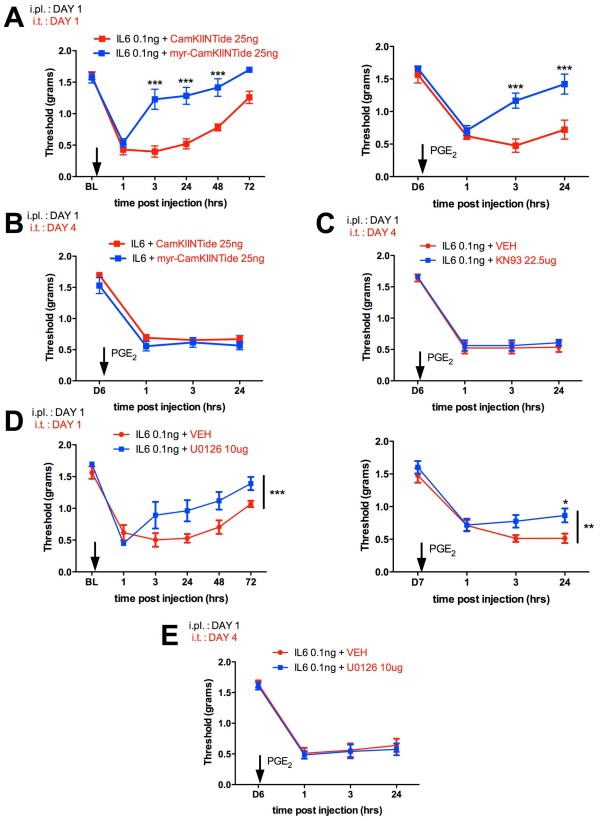
**Lack of involvement of CaMKIIα or MAPK in persistent sensitization. A**) IL-6 was injected into the left hindpaw (i.pl.) of mice and myr-CamKIINTide (peptide CaMKIIα inhibitor) or the non-myristolyated control were injected i.t. at the same time testing the initiation of persistent sensitization. Hindpaw mechanical thresholds were measured at indicated time points. Myr-CamKIINTide inhibited allodynia induced by IL-6 injection (left) and attenuated PGE_2_-precipitated persistent sensitization (right). **B**) On the other hand, Myr-CamKIINTide administered i.t. on day 4 after IL-6 injection had no effect on PGE_2_-precipitated persistent sensitization. **C**) Likewise, no effect was observed in the same experimental paradigm with the small molecule CaMKIIα inhibitor KN93. **D**) IL-6 was injected i.pl. and the MEK inhibitor U0126 or VEH control were injected i.t. at the same time testing the initiation of persistent sensitization. U0126 inhibited allodynia induced by IL-6 injection (left) and attenuated PGE_2_-precipitated persistent sensitization (right). **E**) However, U0126 given i.t. 4 days after IL-6 had no effect on PGE_2_-precipitated persistent sensitization. All experiments N = 6. * p < 0.05, ** p < 0.01, *** p < 0.001, two way ANOVA with Bonferroni post hoc test.

We previously used staurosporine, which inhibits PKC and PKA but not aPKC to demonstrate a specific role for PKMζ in maintenance of persistent sensitization [11]. However, these experiments did not assess a possible role of CaMKIIα or MEK/ERK signaling in initiation or maintenance of persistent sensitization. To test this we used the CaMKIIα peptide inhibitor CamKIINTide in a cell permeable myristoylated (myr) form (or the non-myr peptide as a negative control), the small molecule CaMKIIα inhibitor KN93 and the MEK inhibitor U0126. Importantly, CamKIINTide has been previously reported to reverse late-LTP [23]. Consistent with previous reports suggesting a role of CaMKIIα in the initiation of inflammatory pain states [24,25], myr-CamKIINTide reversed IL-6-induced allodynia when administered intrathecally (i.t.) at the same time as intraplantar (i.pl.) IL-6 (Figure 1A). Moreover, this treatment blocked precipitation of persistent sensitization to PGE_2_ injection into the same hindpaw 6 days later (Figure 1A). Hence, CaMKIIα is involved in the initiation of persistent sensitization. In contrast, when either myr-CamKIINTide (Figure 1B) or KN-93 (Figure 1C) was injected i.t. after the resolution of the initial IL-6-induced allodynia (maintenance phase), neither compound was capable of reversing persistent sensitization revealed by i.pl. PGE_2_ injection. Hence, like protein synthesis inhibitors [11], inhibition of CaMKIIα does not reverse an established, centralized pain state. Identical experiments were conducted with U0126 and, while U0126 was capable of inhibiting initiation of persistent sensitization (Figure 1D), it had no effect on maintenance (Figure 1E). Therefore, we conclude that neither CaMKIIα nor MEK/ERK, but rather a ZIP-reversible process is required for the maintenance of persistent sensitization at dorsal horn synapses.

### BDNF is sufficient to induce persistent sensitization and is required for the initiation and maintenance of persistent sensitization

**Figure 2:**
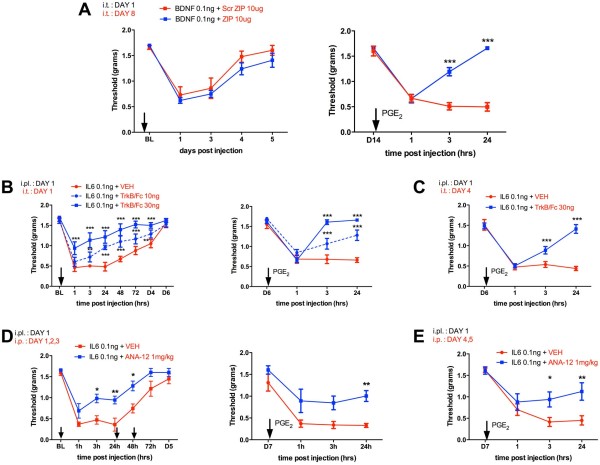
**BDNF is sufficient to establish a ZIP-reversible persistent sensitization and is required for initiation and maintenance of persistent sensitization. A**) BDNF was injected i.t. causing 3 days of allodynia in mice (left) and myristoylated-ZIP or Scr-ZIP were injected i.t. 8 days following BDNF injection. ZIP treatment blocked PGE_2_-precipitated persistent sensitization (right). **B**) IL-6 was injected i.pl. and the BDNF sequestering agent, TrkB/Fc was given i.t. at the same time, dose-dependently blocking IL-6-induced allodynia (left) and PGE_2_-precipitated persistent sensitization (right). **C**) A single i.t. treatment with TrkB/Fc 4 days after i.pl. IL-6 injection was sufficient to attenuate PGE_2_-precipitated persistent sensitization. **D**) The small molecule TrkB antagonist, ANA-12) given i.p. at the same time as i.pl. Il-6 and again 24 and 48 hrs later blocked IL-6-induced allodynia (left) and PGE_2_-precipitated persistent sensitization (right). **E**) Systemic treatment with ANA-12 4 and 5 days after i.pl. IL-6 injection reduced PGE_2_-precipitated persistent sensitization. All experiments N = 6. * p < 0.05, ** p < 0.01, *** p < 0.001, two way ANOVA with Bonferroni post hoc test.

I.t. injection of BDNF is known to induce a long-lasting allodynic state in mice [26] but it is not known if BDNF can induce a ZIP-reversible persistent sensitization as revealed by i.pl. injection of PGE_2_. BDNF administered i.t. induced mechanical allodynia in the hindpaws of mice lasting for at least 3 days and resolving within 5 days (Figure 2A). 8 days following BDNF injection we injected the aPKC inhibitor myr-ZIP (ZIP) or a myr-scrambled peptide (Scr ZIP) i.t.. Because a previous study had suggested that the effects of ZIP may only last for 2 days [27], we waited for 6 days following i.t. injection of ZIP to assess subsequent PGE_2_ precipitated persistent sensitization. Mice that received ZIP on day 8 showed only a transient allodynia following PGE_2_ injection whereas mice receiving Scr ZIP demonstrated at least 24 hrs of allodynia in response to PGE_2_ injection (Figure 2A). Hence BDNF is sufficient to stimulate a ZIP-reversible persistent sensitization.

We then asked whether BDNF sequestration or blockade of TrkB would inhibit IL-6-induced initiation and/or maintenance of persistent sensitization. To test initiation, the BDNF sequestering agent, TrkB/Fc was injected i.t. at the same time as i.pl. IL-6. TrkB/Fc dose-dependently disrupted IL-6-induced allodynia and PGE_2_ precipitated persistent sensitization (Figure 2B). Importantly, when TrkB/Fc was injected i.t. following the resolution of IL-6-induced allodynia, this treatment significantly reversed the maintenance of persistent sensitization (Figure 2C) similar to previous observations with ZIP. If this effect was dependent on BDNF interaction with TrkB, we hypothesized that administration of the small molecule TrkB antagonist ANA-12 should achieve the same effect [28]. ANA-12, which has systemic availability and penetrates the CNS [28], was injected intraperitoneal (i.p.) at the time of IL-6 injection and again 24 and 48 hrs later. This treatment significantly reversed IL-6 induced allodynia and persistent sensitization revealed by PGE_2_ injection on day 7 following IL-6 (Figure 2D). Remarkably, when ANA-12 was given i.p. on day 4 and 5 after i.pl. IL-6 injection and persistent sensitization was precipitated with PGE_2_ on day 7 (a time where ANA-12 should be cleared from the CNS), persistent sensitization was reversed (Figure 2E). Hence, BDNF, acting via trkB, is required for the initiation and maintenance of persistent sensitization.

### BDNF increases PKMζ protein levels and phosphorylation at spinal synapses

**Figure 3:**
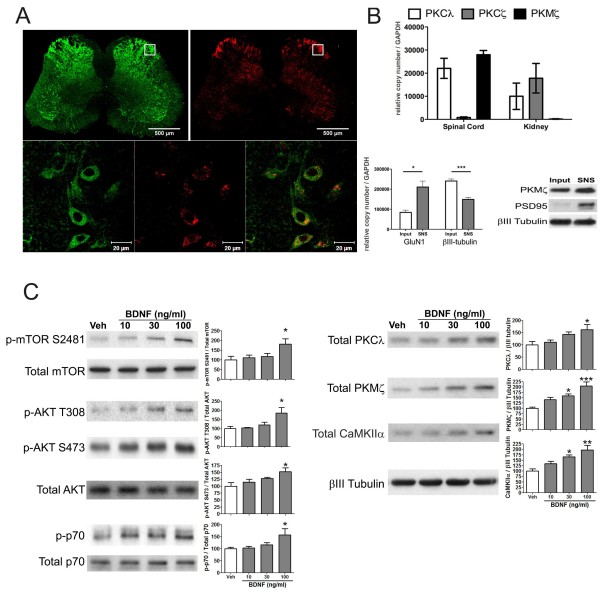
**PKCλ and PKMζ protein localize to central neurons in the dorsal horn and are increased by BDNF treatment.** (**A**) Slices were prepared from mice and stained with an atypical PKC (aPKC) antibody (red) and the neuronal marker (found in soma and post-synaptic sites) SAP-102 (green). The top panel shows that aPKC immunoreactivity is localized primarily to the spinal dorsal horn. Lower panels (from inset) show that aPKC immunoreactivity is localized almost exclusively to neurons in the dorsal horn. (**B**) qPCR was used to assess mRNA expression of aPKC isoforms in spinal cord and kidney (N = 3). Spinal SNSs were prepared and mRNA or protein was isolated. Spinal SNSs were enriched for GluN1 mRNA and were βIII tubulin poor, they were likewise enriched in PSD95 protein as shown by Western blotting compared to equal protein concentration of whole spinal cord homogenate (input). **C**) SNSs were isolated from mouse lumbar spinal cord and exposed to increasing concentrations of BDNF for 15 min. BDNF dose-dependently increased mTOR, AKT and p70S6 kinase (p70) phosphorylation when standardized to total protein levels. BDNF also increased total levels of PKCλ PKMζ and CaMKIIα when compared to loading control βIII tubulin (N = 6). * p < 0.05, *** p < 0.001, one way ANOVA with Bonferroni post hoc test compared to Veh.

Having established a role for BDNF in initiation and maintenance of persistent sensitization, we asked if BDNF regulates PKMζ and/or other aPKCs at spinal synapses. We investigated other aPKCs because it has recently been suggested that PKMζ is not required for the maintenance of late-LTP or long-term memory using genetic knockouts [9,10]. It has also been shown that ZIP blocks PKMζ and PKCλ [10] indicating that ZIP affects all aPKCs. Finally, ZIP still effectively reverses late-LTP and long-term memory in mice lacking PKMζ suggesting a functional redundancy of aPKCs in plasticity pathways [9,10,29]. We first assessed aPKC mRNA expression and protein localization in the mouse spinal cord. As we have shown previously in rat [15], PKCζ mRNA was not expressed in the mouse spinal cord whereas PKCλ and PKMζ were both robustly expressed by qPCR (Figure 3B). Likewise consistent with previous findings in rat [14], aPKC protein localized largely to the dorsal horn of the spinal cord and this immunoreactivity was found exclusively in neurons (Figure 3A inset). Because the immunostaining does not allow for distinguishing between PKMζ and PKCλ we resorted to isolation of synaptoneurosomes (SNSs, [30]) from mouse lumbar spinal cord where PKMζ and PKCλ could be analyzed seperately by Western blot. These SNS preparations were enriched in GluN1 mRNA [31], were βIII-tubulin mRNA poor (Figure 3B, [32]) and at least 10 fold enriched in PSD-95 protein consistent with enrichment of spinal synaptic structures using this technique (Figure 3B).

**Figure 4:**
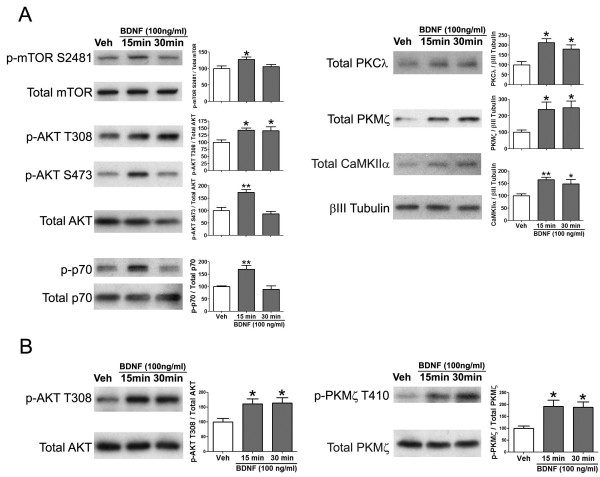
**Time course of BDNF regulation of aPKCs in spinal SNSs.** SNSs were prepared from mice and treated with BDNF for indicated time points. **A**) BDNF transiently increased mTOR, AKT (S473) and p70S6 kinase (p70) phosphorylation but led to a longer lasting increase in AKT phosphorylation at T308 and in total levels of PKCλ, PKMζ and CaMKIIα (N = 6). **B**) To assess whether BDNF increased PKMζ phosphorylation, SNSs were treated with BDNF in the absence of amino acids. BDNF treatment led to a persistent increase in AKT (T308, PDK1 site) and PKMζ (T410, PDK1 site) phosphorylation (N = 6). * p < 0.05, ** p < 0.01, one way ANOVA with Bonferroni post hoc test compared to Veh.

To determine if BDNF regulates aPKC protein levels at spinal synapses we stimulated SNSs with increasing concentrations of BDNF. Because previous studies have suggested a role for mTOR in regulating PKMζ formation in LTP [33] and because BDNF is known to regulate mTOR in hippocampus [34], we also assessed signaling components of the mTOR pathway in these experiments. BDNF increased mTOR S2481 phosphorylation consistent with activation of mTORC2 [35] at spinal synapses with BDNF (Figure 3C). Likewise, BDNF increased AKT phosphorylation at T308 (PDK1 site) and S473 (mTORC2 site) and BDNF increased phosphorylation of the mTORC1 target Thr389 residue on p70 S6 Kinase (p70, [36] Figure 3C). Consistent with engagement of mTORC1-dependent protein synthesis, PKCλ, PKMζ and CaMKIIα protein levels were also increased by BDNF in spinal SNSs (Figure 3C). These effects were time dependent with changes in phosphorylation occurring largely at 15 min of BDNF stimulation and resolving by 30 min. The exception was T308 phosphorylation of AKT (PDK1 site), which persisted for the full 30 min of BDNF exposure (Figure 4A). We also observed long-lasting changes in total amounts of PKCλ, PKMζ and CaMKIIα (Figure 4A), again consistent with a protein synthesis-dependent process. These effects are likely not due to aPKC regulation in sensory afferent terminals because exposure of sensory neurons in culture to BDNF led to robust activation of AKT without any corresponding change in aPKC levels (data not shown).

Because total levels of PKMζ were changed by BDNF exposure to SNSs, we performed experiments where protein synthesis could not occur to assess whether BDNF also changed PKMζ phosphorylation in a persistent fashion. In the absence of amino acids, BDNF failed to increase total PKMζ level in spinal SNSs, however, under these conditions, BDNF robustly increased AKT T308 and PKMζ T410 phosphorylation (Figure 4B). Because both of these phospho sites are acceptors for PDK1 activity these findings suggest that BDNF stimulates PDK1 to achieve persistent increases in downstream target phosphorylation. Hence, BDNF persistently increases PKMζ protein levels and phosphorylation at spinal synapses.

### BDNF stimulates eIF4F complex formation and aPKC nascent synthesis at spinal synapses

**Figure 5:**
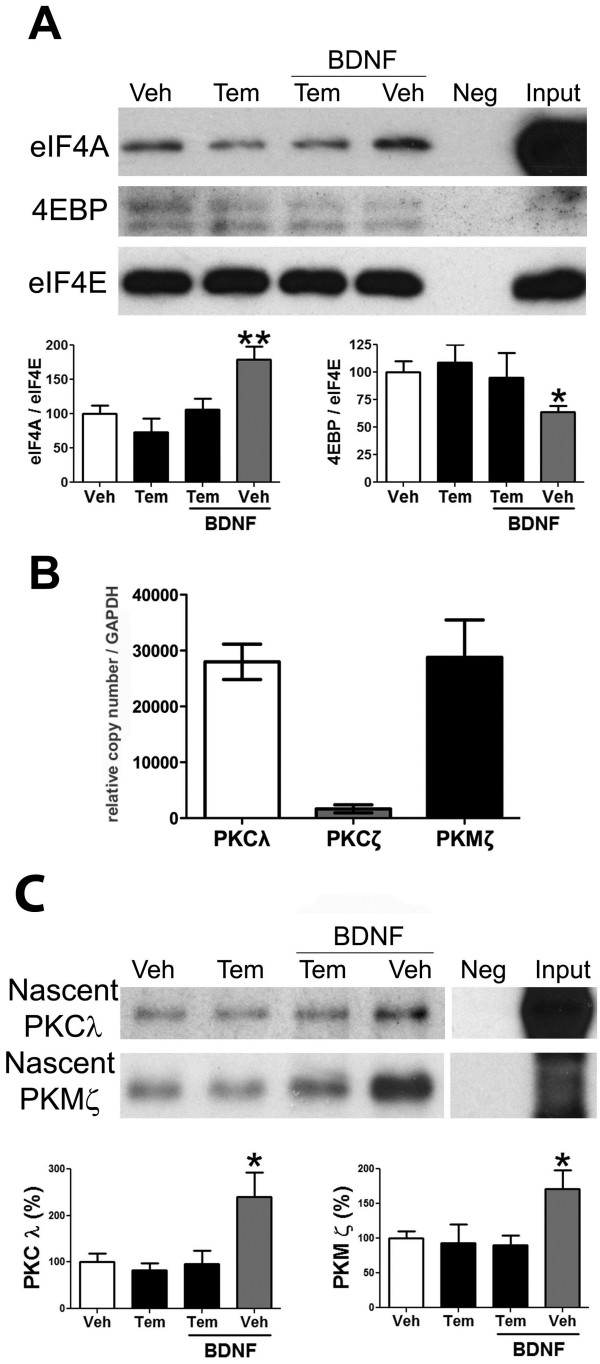
**BDNF enhances eIF4F complex formation and nascent synthesis of PKCλ and PKMζ in spinal SNSs in an mTOR-dependent fashion.** SNSs were prepared from mouse lumbar spinal cord and treated with or without BDNF (100 ng/ml) or the mTORC1 inhibitor temsirolimus (Tem, 100 nM). **A**) Following 15 min treatment with the indicated conditions, 5′m7-GTP pulldown assays were performed and eIF4A, 4EBP and eIF4E were assessed by Western blot. BDNF treatment led to an increase in eIF4A pulldown and a decrease in 4EBP pulldown in an mTORC1-dependent fashion (N = 6). **B**) SNSs were assessed for aPKC mRNA expression by qPCR. SNSs contained PKMζ and PKCλ mRNA but not PKCζ (N = 3). **C**) SNSs were treated with BDNF +/− Tem in the presence of AHA to tag nascently synthesized proteins for 30 min. AHA aPKC was immunoprecipitated and AHA was labeled with biotin using click-chemistry. BDNF treatment led to an increase in nascently synthesized PKCλ and PKMζ in an mTORC1-dependent fashion (N = 6). * p < 0.05, ** p < 0.01, one way ANOVA with Bonferroni post hoc test compared to Veh.

The results presented above suggest that aPKCs are synthesized as a result of BDNF action on spinal synapses. To pursue this idea with more rigor, we first asked if BDNF increases formation of the 5^′^ cap binding complex composed of eIF4E, eIF4A and eIF4G, called eIF4F, at spinal synapses. This complex is involved in promoting cap-dependent protein synthesis and occurs downstream of mTORC1 activation [37]. Using m7-GTP beads, we performed 5^′^ cap pulldown assays on SNSs stimulated with BDNF for 15 min. BDNF increased eIF4A pulldown and decreased 4EBP association with eIF4E, consistent with BDNF inducing formation of the eIF4F complex at spinal synapses (Figure 5A). This effect was completely blocked by inclusion of temsirolimus indicating that BDNF promotes eIF4F complex formation in an mTORC1-dependent fashion (Figure 5A).

We next asked if BDNF increases nascent synthesis of aPKCs in an mTORC1-dependent fashion. To do this, we first assessed whether aPKC mRNA was found at spinal synapses. SNSs were prepared and mRNA levels were assessed by qPCR. PKMζ and PKCλ, but not PKCζ, mRNA was detected in spinal SNSs demonstrating that these SNSs are capable of supporting nascent synthesis of PKCλ and PKMζ and supporting the notion that PKCλ and PKMζ mRNAs are transported to synapses in the dorsal horn (Figure 5B). Having established that PKCλ and PKMζ mRNA are found at synapses, we used azidohomoalanine (AHA), a click-chemistry compatible methionine analogue that does not interfere with other cellular processes [38], to assess nascent synthesis of PKCλ and PKMζ. The methionine stores were depleted in spinal SNSs by inclublting them in methionine free media for 15 min. This was followed by stimulation of the SNSs with BDNF in the presence of AHA for 30 min. aPKC proteins were immunoprecipitated and labeled with biotin using click-chemistry to label only proteins that had incorporated AHA (e.g. nascently synthesized proteins). Remarkably, BDNF led to a robust increase in nascently synthesized PKCλ and PKMζ that was completely abrogated by mTORC1 inhibition (Figure 5C). Hence, BDNF induces PKCλ and PKMζ nascent synthesis via an increase in eIF4F complex formation downstream of mTORC1 activation at spinal synaptic structures.

### BDNF increases mTORC1 activity and aPKC formation at cortical synapses

**Figure 6:**
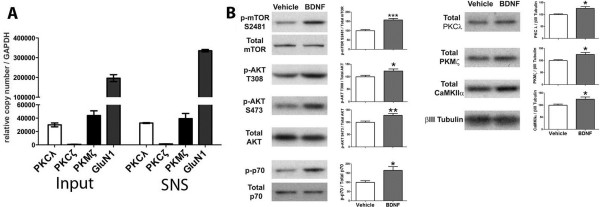
**BDNF activates mTOR and increases PKCλ, PKMζ and CaMKIIα protein in cortical SNSs. A**) qPCR was used to assess aPKC mRNA expression in whole cortex samples (Input) and in cortical SNSs SNSs. PKMζ and PKCλ mRNA were found in cortex and cortical SNSs but not PKCζ. GluN1 mRNA was enriched in cortical SNSs (N = 3). **B**) SNSs were prepared from mouse cortex and stimulated with 100 ng/ml BDNF for 15 min. Treatment led to an increase in mTOR, AKT and p70S6 kinase (p70) phosphorylation as well as increased levels of total PKCλ, PKMζ and CaMKIIα (N = 6). * p < 0.05, ** p < 0.01, *** p < 0.001, student’s t-test.

**Figure 7:**
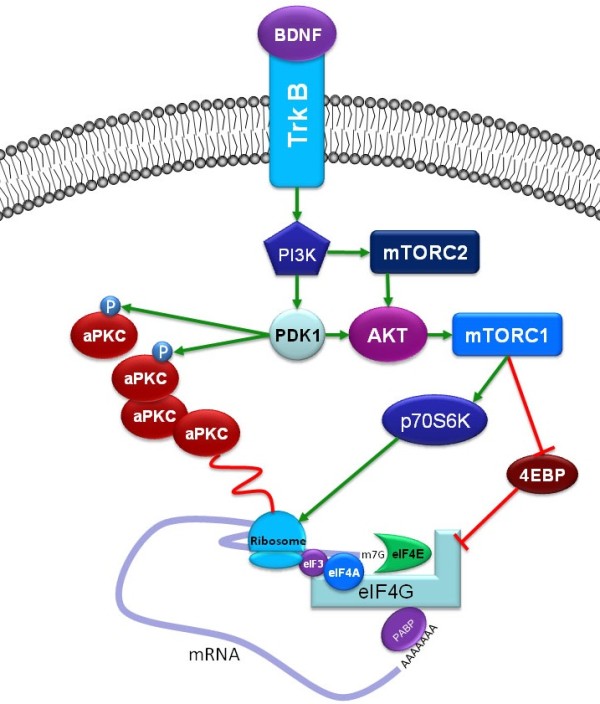
**BDNF regulation of aPKCs at central synapses.** Summary cartoon of major findings presented here. At spinal synapses, BDNF activated trkB leading to increased PI3K and PDK1 activity. This stimulates aPKC phosphorylation at T410 and an increase in AKT activity thereby stimulating mTORC1. This increase in mTORC1 activity leads to an increase in eIF4F complex formation resulting in an enhancement of aPKC translation at spinal synapses. Data from cortical SNS preparations strongly suggest that this signaling complex engaged by BDNF resulting in regulation of PKMζ is present across central synapses.

Having shown that BDNF regulates PKCλ and PKMζ formation in an mTORC1-dependent fashion at spinal synapses we then asked it BDNF also achieves similar effects at cortical synapses where both BDNF and PKMζ (and possibly PKCλ [9,10,29]) are known to play an important role in LTP and long-term memory maintenance [8,16]. By qPCR, PKCλ and PKMζ mRNA localized to cortical SNSs as shown above for spinal SNSs and these cortical SNSs were also enriched for GluN1 mRNA (Figure 6A). Likewise identical to observations in spinal SNSs, BDNF stimulated an increase in mTOR S2481, AKT T308 and S473 and p70 phosphorylation (Figure 6B). BDNF also increased CaMKIIα, as shown previously [39], and PKCλ and PKMζ protein levels (Figure 6B). Hence, BDNF regulation of PKMζ formation is conserved across CNS synapses (Figure 7).

## Discussion

While PKMζ is well-recognized as a potential molecular mechanism for the maintenance of LTP and long-term memory [8,40] and its important role in pain plasticity has recently been elucidated [11-15], neurotransmitter systems involved in the regulation of PKMζ have not been described in detail. Moreover, the specific role of PKMζ in CNS plasticity has recently been called into question with PKCλ emerging as a potential redundant mechanism in CNS plasticity [9,10,29]. Here we demonstrate that BDNF promotes persistent sensitization via a ZIP-reversible mechanism. Moreover, we show that BDNF is critical for both the initiation and maintenance of persistent sensitization, a role that it may uniquely share with an aPKC-dependent process [11,13]. Linked to these *in vivo* findings, we further demonstrate that BDNF regulates PKCλ and PKMζ synthesis via an mTORC1-dependent pathway and PKMζ phosphorylation via PDK1 at spinal and cortical synapses. Importantly, we show definitively, for the first time, that both PKCλ and PKMζ are synthesized in an activity-dependent fashion at synaptic sites. Therefore, BDNF plays a key role in regulating aPKCs in the pain pathway elucidating a hitherto unrecognized pathway regulating the maintenance of a centralized chronic pain state.

PKMζ is an atypical PKC that was first recognized as a constitutively active kinase that may play a role in maintenance of late-LTP [8,41]. Because PKMζ lacks a regulatory region, once translated, and phosphorylated by PDK1, the kinase has the potential to maintain autonomous activity over extended periods of time, satisfying theoretical considerations for a kinase-mediated mechanism maintaining late-LTP [8]. This hypothesis has been borne-out by a body of subsequent work demonstrating a key role for PKMζ in maintaining late-LTP and also long-term memory [42,43]. While parallels between molecular mechanisms of long-term memory and pain plasticity have long been recognized, only recently has PKMζ been elucidated as a potential target for maintenance of chronic pain states. PKMζ appears to play different roles in different anatomical locations in the pain pathway. PKMζ in sensory neurons is important for nerve growth-factor mediated hyperexcitability [44]. PKMζ in the anterior cingulate cortex plays a key role in regulating tonic-aversive aspects of chronic neuropathic pain [12,15]. Interestingly, a ZIP-reversible process in the spinal cord appears to play little, if any role in maintaining chronic neuropathic pain [12,15], perhaps because this chronic pain state is critically dependent on ongoing afferent input to the spinal dorsal horn [45]. In contrast, in chronic pain states wherein afferent input resolves but hypersensitivity either persists or can be rekindled by a normally subthreshold stimulus (e.g. persistent sensitization [11], CFA-induced inflammation [22] or chronic post ischemic pain [13,46]) the maintenance of this pain state is reversed by spinal injection of ZIP. Our present findings expand on these previous results demonstrating that while CaMKIIα and MEK/ERK signaling is required for initiation of persistent sensitization, these kinases do not play an active role in the maintenance phase of persistent sensitization. These findings can be viewed as in contrast to other models, such as CFA, formalin, and/or incision, wherein ERK [47,48] and CaMKIIα [24] play an important role in initiation and maintenance of a continuous hypersensitive pain state. Such differences, as mentioned above, may be related to afferent input engaged by these stimuli, which presumably resolves during the maintenance phase of the persistent sensitization model. We feel it is important to point out that i.t. drug applications during the maintenance phase are made when the mice show no overt signs of mechanical hypersensitivity. If these compounds were to be given at the same time as PGE_2_ injection an inhibitory effect might be expected because afferent input would be re-engaged, likely utilizing priming-dependent peripheral mechanisms that have recently been elucidated [49]. These results, combined with our previous findings, strongly implicate aPKCs as the sole family of kinases responsible for the maintenance of persistent sensitization.

Despite the emerging role of PKMζ and potentially PKCλ in pain plasticity, mechanisms involved in aPKC regulation in the pain pathway are nearly completely unknown. We hypothesized that BDNF might play a key role in regulating aPKCs. This hypothesis was based on a known role of BDNF in pain states consistent [18-20] with the known effects consistent with an involvement of aPKCs. While BDNF can have several sources in the spinal dorsal horn, acutely it is released from nociceptors synapsing in the outer lamina of the dorsal horn [17,19] where it regulates inflammatory but not neuropathic pain [19]. BDNF also plays an important role in regulating LTP at dorsal horn synapses [20] consistent with the known role of BDNF in LTP in other CNS regions [16]. These findings, combined with our present results, are consistent with a model wherein BDNF released from nociceptive endings in the spinal dorsal horn initiates signaling cascades that lead to the formation and phosphorylation of aPKCs at these synapses. Although spinal BDNF plays a role in neuropathic pain, as mentioned below, this has been linked to release from microglia [50,51] and not nociceptor terminals because neuropathic pain develops normally in mice lacking BDNF expression in nociceptors [19]. This finding is consistent with previous findings showing a limited role of a spinal ZIP-reversible process in neuropathic pain. We cannot, however, rule out an effect of microglial BDNF in our experiments. In that regard, BDNF is also known to play an important role in microglial activity [21] and neuropathic pain where it regulates GABAergic modulation of spinal circuits through disruption of Cl^-^ homeostasis [51,52]. Interestingly, this mechanism appears to be shared in morphine-induced hyperalgesia [53]. Our findings from spinal SNS experiments clearly demonstrate that BDNF applied exogenously is capable of stimulating synthesis of PKCλ and PKMζ and phosphorylation of PKMζ. Whether BDNF released from microglia is incapable of achieving these effects at spinal synapses will have to await further experimentation. Although BDNF can have trkB-independent actions [54], we surmise that the effects of BDNF in our experiments were mediated by trkB due to the effect of the trkB antagonist ANA-12 [28].

An important implication of our current findings is that BDNF not only plays a role in initiating a centralized chronic pain state but that it also plays an active role in maintaining such a pain state via regulation of aPKCs. If this is the case, what is the source of BDNF? It is unlikely to be derived from presynaptic release from nociceptors because these sensory neurons are unlikely to be active after the resolution of IL-6-induced allodynia. It is also unlikely that microglia are the source because this would be inconsistent with the neuropathic pain findings for ZIP [12,13,15]. Important clues might be gleamed from the LTP literature wherein both pre- and post-synaptic release of BDNF regulates consolidation of late-LTP [55-57]. Interestingly, this likely involves alternatively spliced isoforms of BDNF in hippocampus [58] facilitating the possible recognition of such a mechanism being engaged in the spinal dorsal horn. While these experiments are outside of the scope of the present findings, this is likely to be a fruitful area of future research to gain a better understanding of maintenance mechanisms of a centralized chronic pain state.

Another important question raised by our findings relates to the dependence of maintenance of persistent sensitization on aPKCs but not protein synthesis. If BDNF regulates both PKCλ and PKMζ synthesis and PKMζ phosphorylation and initiation and maintenance of persistent sensitization are dependent on both aPKCs and BDNF but only initiation is dependent on protein synthesis, how is this seeming contradiction resolved? One possible explanation is that in the absence of protein synthesis, BDNF regulation of PKMζ phosphorylation is sufficient to maintain the chronic pain state. Interestingly, in spinal SNSs, BDNF stimulation of mTORC1 activity was transient whereas PDK1 mediated phosphorylation of both AKT and PKMζ was persistent. Hence, it is physiologically feasible that in the absence of protein synthesis, BDNF-mediated phosphorylation of PKMζ is sufficient to maintain persistent sensitization. Another possibility is that PKMζ, and possibly PKCλ, has an exceptionally long half-life at synapses. In this scenario, despite blockade of protein synthesis over long periods, aPKCs formed via previous protein synthesis would be capable of overcoming a lack of new protein availability due to its long half-life. Our preliminary observations (Melemedjian, Ghosh and Price, unpublished observations) support this model but ultimately require further experimentation. However, it is clear that BDNF can maintain late-LTP when protein synthesis is inhibited via a PKMζ-dependent mechanism [59] suggesting that similar mechanisms may be at play in the setting of persistent sensitization.

Importantly, we demonstrate that BDNF regulates aPKC formation in cortical SNSs in an analogous fashion to spinal SNSs. Insofar as both the maintenance of a centralized chronic pain state and long-term memory require both BDNF [60] and PKMζ [8], and considering that we demonstrate that BDNF regulates aPKCs across CNS structures, this illustrates the potential existence of a conserved pathway for the maintenance of synaptic plasticity from pain to memory. We propose that this has profound implications for understanding how mechanisms of plasticity evolved in central nervous systems and we suggest that these mechanisms might have first evolved for the most rudimentary neural function: protecting the organism against potentially lethal tissue injury. An important point moving forward will be to unveil how different aPKC isoforms contribute to pain plasticity through genetic models, as we have recently reviewed [29]. This need is highlighted by the recent findings from the learning and memory literature showing that genetic removal of PKMζ fails to affect learning and memory despite the continued efficacy of ZIP in these animals, suggesting a potential redundant role of PKCλ in these pathways [9,10]. It will be crucial to carefully examine the role of PKCλ in plasticity moving ahead.

In closing, we reveal that BDNF regulates the formation of PKCλ and PKMζ and phosphorylation of PKMζ and that BDNF/aPKC signaling forms a signaling axis required for the maintenance of a centralized chronic pain state. Our results imply that spinally directed therapeutics targeting BNDF and/or aPKCs might offer disease-modifying effects on certain chronic pain states in humans that are currently only treated by palliative management. The generation of such a class of therapeutics would have profound implications for the treatment of chronic pain.

## Methods and materials

### Experimental animals

All animal procedures were approved by the Institutional Animal Care and Use Committee of The University of Arizona and were in accordance with International Association for the Study of Pain guidelines. Male ICR mice (20–25 g; Harlan) were used for all studies.

### Mechanical testing

Animals were treated as described previously [11]. In brief, animals were placed in acrylic boxes with wire mesh floors, and baseline mechanical withdrawal thresholds of the left hindpaw were measured after habituation for 1 h using the up-down method [61]. The experimenter making measurements was always blinded to the experimental conditions. For day 1 experiments with IL-6, IL-6 was injected into the plantar surface of the left hindpaw in a volume of 25 μl. For day 1 experiments with BDNF, BDNF was injected intrathecally (i.t.) in a volume of 5 μl. For intrathecal treatments on day 1, drugs were injected immediately after intraplantar (i.pl.) injections under brief (<3 min) isoflurane anesthesia in a volume of 5 μl [62]. For day 1 experiments with ANA-12, ANA-12 was injected intra-peritonially (i.p.) on day 0, 1 and 2 following IL-6 injection. For experiments with intrathecal treatments on day 4 or later, mice were tested before i.t. injection to assure that allodynia had completely resolved. I.T. injections were done at the indicated time points under isoflurane anesthesia as described above. For day 4 experiments with ANA-12, ANA-12 was injected i.p. on day 4 and 5 following IL-6 injection. PGE_2_ (100 ng) was injected on day 6 or later in the plantar surface of the left hindpaw in a volume of 25 μl. Allodynia testing was then done at the time points indicated in the text.

### PCR

Total RNA was extracted from tissue and synaptosomal preparations the RNeasy mini kit (#74104 QIAGEN, Valencia, CA, USA) according to the manufacturer’s instructions. RNA quantification and purity were tested using a Nanodrop® spectrophotometer. 1 μg of total RNA was used for cDNA synthesis with iScript™ Reverse Transcription Supermix for RT-qPCR kit (#170-8890 Biorad). RT-PCR reactions were performed on an ABI 7500 Fast Real-time PCR System with SYBR Green PCR master mix (#4309155 Applied Biosystems, Life Technologies, Carlsbad, CA, USA) using default two-step (95°–60°) amplification. All primer pairs were tested by running 3–4 fold dilution across at least 5 dilution points. Primers only passed if they had a calculated efficiency between 97–103% with an R^2^ value greater than 0.98 and had a single, shoulder-free peak upon melt curve analysis. Primer sequences are given in Table 1. Reactions were run in triplicate; measurements are based on at least three independent samples. No-RT and Cq dilution controls were routinely performed to check for genomic DNA and inhibitory contamination respectively. Melt curves were performed with each run to insure specific amplification products. Each reaction was normalized to the expression of glyceraldehyde 3-phosphate dehydrogenase (GAPDH). Expression numbers given in the paper were calculated by arbitrarily assigning GAPDH a value of 2^20^ and calculating the expression relative to GAPDH. GAPDH-normalized values were compared with normalization to Eef1A and Rpl29 to ensure controls and comparative data were consistent (data not shown).

**Table 1:** Primer sequences used for qPCR experiments

**Gene**	**Forward primer**	**Reverse primer**	**Size**
GAPDH	AGGTCGGTGTGAACGGATTTG	GGGGTCGTTGATGGCAACA	95
βIII Tubulin	TAGACCCCAGCGGCAACTAT	GTTCCAGGTTCCAAGTCCACC	127
PKCλ	CCATGTGTACCAGAGCGTCCT	TGTGGCCATTTGCACAATACA	106
PKCζ	CAGGGACGAAGTGCTCATCA	CACGGCGGTAGATGGACTTG	95
PKMζ	AGCAGAGAAAGCCGAGTCCA	TTAAAGCGCTTGGCTTGGAA	96
GluN1	ATGCACCTGCTGACATTCG	TATTGGCCTGGTTTACTGCCT	142

### Synaptoneurosome preparation and treatment

Spinal cord and cortical synaptoneurosomes (SNS) were prepared from 3-weeks-old male ICR mice as previously described [30]. Briefly, dissected spinal cords or cortices were homogenized at on ice in homogenization buffer (in mM) 118 NaCl, 4.7 KCl, 1.2 MgSO_4_, 2.5 CaCl_2_ and 1.53 KH2PO_4_, 212.7 glucose pH 7.4], supplemented with Complete protease inhibitors (Sigma, St. Louis, MO) and 40 U/ml recombinant human RNase inhibitor (Life technologies, Grand Island, NY). Samples were successively filtered through three layers of 100 μm and 11 μm nylon mesh filters (Millipore, Bedford, MA) and centrifuged at 1000 × g for 20 min. The pelleted SNS were suspended in DMEM/F12 (Life technologies, Grand Island, NY) tissue culture media supplemented with Complete protease inhibitors and RNase inhibitor. Some experiments were carried out in homogenization buffer to prevent protein synthesis since this buffer does not contain amino acid. The resuspened SNS were then treated with various concetrations of BDNF (R&D Systems, Minneapolis, MN) for 15 or 30 min at 37°C. SNS were centrifuged at 20000 × g for 2 min, the pellet was resuspended in lysis buffer (50 mM Tris HCl, 1% Triton X-100, 150 mM NaCl, and 1 mM EDTA at pH 7.4), ultrasonicated and centrifuged at 20000 × g for 15 min. The supernatant was collected and assayed using Western blot analysis.

### Nascent aPKC synthesis assay

SNS were suspended in methionine-free media (cat # 21013–024, Life technologies, Grand Island, NY) and pretreated with vehicle or temsirolimus (100 nM, LC Labs, Woburn, MA) for 15 min at 37°C. Azidohomoalanine (AHA) is a methionine analogue that cells can incorporate into nascentlly synthesized protein. AHA (50 μM, Life technologies, Grand Island, NY) was added to the SNS suspension and incubated at 37°C for 30 min. SNS were then centrifuged at 20000 × g for 2 min and lysis buffer was added to the pellet. Protein was extracted by ultrasonication, centrifugation at 20000 × g for 15 min and collection of the supernatant. PKMζ was immunoprecipitated by incubating the supernatant with 1:50 mouse anti-PKCζ antibody (cat # sc-17781, Santa Cruz Biotechnology, Santa Cruz, CA) overnight at 4°C. The samples where then incubated with protein G sepharose beads (Sigma, St. Louis, MO) for 3 hr at 4°C, followed by centrifugation and wash with lysis buffer 3 times. The pelleted beads were suspended in Tris-SDS buffer (1% SDS and 50 mM Tris–HCl, pH 8.0), centrifuged and the supernatant was collected. At this stage, the supernatant contains the immunoprecipitated PKMζ where the nascently synthesized form would have incorporated AHA. AHA was biotinylated using Click-it Protein Analysis Detection Kit (Life technologies, Grand Island, NY) according to the manufacturer’s instructions. The biotinylated PKMζ was detected by Western blotting.

### 5^′^m7-GTP pulldown assays

After the protein extraction, 50 μg protein was incubated with 7- methyl GTP Sepharose 4B beads (GE Healthcare) in the presence of 100uM GTP for 2 h at 4°C. Unconjugated sepharose 4B beads were used for the negative controls. The beads were then pelleted and washed twice with lysis buffer. eIF4E, eIF4A and 4EBP bound to the precipitated beads were analyzed by western blotting.

### Western blotting

Fifteen micrograms of protein in 1X Laemmli Sample Buffer containing 5% v/v β-mercaptoethanol were loaded in each well and separated by standard 10% SDS-PAGE. Proteins were transferred to Immobilon-P membranes (Millipore, Billerica, MA) and then blocked with 5% dry milk for 3 h at room temperature. The blots were incubated with primary antibody overnight at 4°C and detected the following day with donkey anti-rabbit antibody conjugated to horseradish peroxidase (Jackson Immunoresearch, West Grove, PA). Signal was detected by ECL on chemiluminescent films. PKCλ and PKMζ were recognized by a pan-aPKC antibody and differentiated by size. Since PKCζ mRNA was not present in these tissues, the presence of that protein, the only other member of the aPKC family, was excluded. Each phosphoprotein was normalized to the expression of the corresponding total protein on the same membrane. The p-PKMζ antibody does not recognize p-PKCλ and therefore could not be used to determine phosphorylation of PKCλ. This antibody does recognize p-PKCζ but consistent with an absence of PKCζ in these tissues, no band was observed at the appropriate size for that protein with the p-PKC/Mζ antibody. Densitometric analyses were performed with Image J software (NIH, Bethesda, MD) using the gel analysis tool available as a plugin from McMaster University on the following website: macbiophotonics.ca. Densitometry was done following instructions given for this plugin for ImageJ.

### Immunohistochemistry (IHC)

IHC on mouse spinal cord was done as described previously on fresh frozen 20 μm sections of mouse lumbar spinal cord [63]. Localization of aPKC was assessed with the Santa Cruz sc-216 antibody and SAP-102 (Cell Signaling Technologies) was used to label neuronal structures.

### Primary antibodies and chemicals

The following rabbit antibodies were obtained from Cell Signaling (Danvers, MA): p-AKT (Ser473, cat# 4058 and Thr308 cat# 2965), total AKT (cat# 4691), p-mTOR (Ser2481, cat# 2974), total mTOR (cat# 2983), p-p70 (Thr389, cat# 9205), total p70 (cat# 9202), p-PKC/Mζ (Thr410, cat# 9378), CaMKIIα (cat# 3357), eIF4A (cat# 2424), 4EBP1/2 (cat# 9452 and 2845) and eIF4E (cat# 9742). Total rabbit aPKC was from Santa Cruz Biotechnologies (cat# sc-216, Santa Cruz, CA) and βIII-Tubulin was from Promega (cat# G7121, Madison, WI). Human recombinant IL-6, BDNF and TrkB/Fc were from R&D Systems; myristoyalated CamKIINTide, CamKIINTide and KN-93 were from Calbiochem; ANA-12 was from Maybridge; UO126 was from Tocris Bioscience; and prostaglandin E_2_ (PGE_2_) was from Cayman Chemical Company. Stock solutions of IL-6, CamKIINTide, KN-93, ANA-12 and UO126 were made in 100% DMSO. BDNF stock solution was made in sterile PBS containing 0.1% BSA and TrkB/Fc stock solution was made in sterile PBS. PGE_2_ stock solutions were made in 100% ethanol. All drugs except U0126 and ANA-12 were diluted to final concentrations in saline for injection. U0126 was diluted to final concentration in 45% cyclodextrin. ANA-12 was diluted to final concentration in 10% polyethylene glycol 300. Matching vehicles (saline + matching amount of stock diluent except for U0126 and ANA-12 where saline with 45% cyclodextrin or saline with 10% polyethylene glycol 300, respectively) were used as a control in all experiments.

## Competing interests

The authors declare that they have no competing interests.

## Authors’ contributions

TJP, OKM and SG conceived of the study and designed experiments, OKM, DVT, MNA, EKM, JKM, VMB and CJT performed experiments, OKM, DVT, MNA, EKM, SG and TJP analyzed data, OKM, SG and TJP wrote the manuscript. All authors read and approved the final manuscript.
